# The Association of Body Mass Index and Waist Circumference with Sepsis-Related Mortality in South Korea

**DOI:** 10.3390/diagnostics14060574

**Published:** 2024-03-07

**Authors:** Tak-Kyu Oh, In-Ae Song

**Affiliations:** 1 Department of Anesthesiology and Pain Medicine, Seoul National University Bundang Hospital, Seongnam 13620, Republic of Korea; airohtak@hotmail.com; 2 Department of Anesthesiology and Pain Medicine, College of Medicine, Seoul National University, Seoul 01811, Republic of Korea

**Keywords:** sepsis, obesity, waist circumference, mortality, body mass index

## Abstract

Obesity is a major public health problem worldwide and is associated with increased morbidity and mortality. However, studies have shown that obesity has sepsis-related mortality benefits. We aimed to determine whether there is an improved sepsis-related survival rate in patients with obesity in South Korea. We included data from 77,810 adults with sepsis between 1 January 2013 and 31 December 2020, extracted from the National Health Insurance Service database in South Korea. The patients underwent standard health examinations within a year before sepsis, and body mass index (BMI) and waist circumference (WC) were used to reflect obesity. Lower 30-day and 1-year mortality rates were observed in the overweight and obesity groups after adjusting for confounders, including WC. However, there was no difference in mortality between the patients with severe obesity and those with normal BMI. Underweight was associated with higher 30-day and 1-year mortality. Higher 30-day and 1-year mortality was found in the high and very high WC groups. In conclusion, patients with abdominal obesity and overweight and obesity and with sepsis showed reduced mortality, whereas underweight patients with sepsis showed increased mortality in South Korea.

## 1. Introduction

Sepsis is defined as a life-threatening organ dysfunction caused by a dysregulated host response to infection [1]. Sepsis and septic shock are the leading causes of mortality worldwide, representing major public health burdens. The global age-standardized incidence of sepsis is 678 cases per 100,000. In addition, the global age-standardized mortality for sepsis in 2017 was 148 deaths per 100,000 [2]. Septic shock has the highest mortality rate of approximately 50% [3].

Obesity is a major public health problem and is associated with chronic diseases such as cardiovascular disease, diabetes mellitus, hypertension, and all-cause mortality in adults [4]. However, the obesity paradox—lower sepsis-related mortality in obese patients—has been reported in many studies despite the well-established harmful effects of obesity [5,6]. The results of studies on the obesity paradox of sepsis-related mortality were mixed [7]. Meta-analyses have found that obesity reduced sepsis-related mortality in patients admitted to intensive care units (ICUs) [8,9]. However, each study included and analyzed in these meta-analyses showed controversial results regarding this paradox [10,11,12,13,14,15].

One reason for these mixed results may be that the definition of obesity varied among these studies. Most studies have defined obesity by BMI, and few have defined it by waist circumference (WC) [16]. WC, which is considered a rough estimate of abdominal obesity, is associated with cardiovascular complications and mortality. WC is also associated with visceral fat and insulin resistance. Obesity is also defined differently according to ethnicity. Asians have a higher cardiometabolic risk at a lower BMI or WC than other populations [17].

In a small single-center retrospective study in South Korea, the underweight group (BMI < 18.5 kg/m^2^) had significantly higher mortality than the normal weight group (18.5 ≤ BMI < 25 kg/m^2^) [18]. However, a decrease in mortality was not observed in the obese group (BMI ≥ 25 kg/m^2^). Nevertheless, the relationship between obesity and sepsis-related mortality has not yet been identified.

Therefore, we aimed to investigate whether the obesity paradox could be observed in sepsis-related mortality and to define obesity by both BMI and WC measured in biannual standardized health examinations.

## 2. Materials and Methods

### 2.1. Study Design and Ethical Statements

This retrospective cohort study used the national database in South Korea and followed the Strengthening the Reporting of Observational Studies in Epidemiology guidelines [19] This study was approved by the Institutional Review Board (X-2201-735-902) and the National Health Insurance System (NHIS) (NHIS-2022-1-337). The requirement for informed consent was waived owing to the retrospective design of this study and the use of anonymous data extracted from the NHIS database of the Republic of Korea.

### 2.2. Data Source

The database used in this study was based on the NHIS, a single public health insurer in the Republic of Korea that manages databases on the diagnoses of diseases and prescriptions for procedures and medication. Diseases are diagnosed and registered by physicians using the International Classification of Diseases (ICD-10) and External 10th revision codes. The NHIS database also includes the demographic and socioeconomic status of patients. NHIS data contain information on almost all patients because the Korean national healthcare system covers all residents within the territory of the Republic of Korea, except for beneficiaries of medical aid.

### 2.3. Study Population

The inclusion criterion in this study was adults (≥18 years) who were admitted to hospitals with diagnoses of sepsis or septic shock from 1 January 2016 to 31 December 2020. ICD-10 codes (A40, A41, and R65.2) were used to identify cases of sepsis and septic shock.

Patients who did not undergo standardized health examinations in a year prior to sepsis were excluded because data on BMI and WC were not available. Multiple hospital admissions (≥2) associated with sepsis or septic shock were also excluded to focus on the most recent related admissions.

In South Korea, NHIS subscribers aged ≥ 40 years are recommended to undergo standardized health examinations every 2 years, and expenses for standardized health examinations are covered by the government [20]. Therefore, 50% of all adult individuals aged ≥ 40 years might be recommended to undergo standardized health examinations in a certain year (i.e., the year prior to sepsis). Moreover, a recent study has reported a 70–80% participation rate in standardized health examinations in South Korea despite variations in age, disability, and socioeconomic status [21].

### 2.4. BMI and WC (Independent Variable)

BMI and WC data, which were derived from standardized health examinations in a year prior to sepsis, were used in this study. Therefore, the duration from the date of BMI/WC measurement to sepsis onset could have ranged between 1 day and 2 years. BMI was calculated using body weight (kg) and body height (cm) of the study population, whereas WC was measured at the midpoint between the lower end of the last rib palpated at the axillary midline and upper part of the iliac crest. The participants underwent measurement in a state of exhalation in the standing position; the tape was level with the floor and did not press against the skin.

BMI was categorized into five groups (<18.5, 18.5–24.9, 25.0–29.9, 30.0–34.9, and >35.0 kg/m^2^ (underweight, normal, overweight, obesity, and severe obesity, respectively)) based on the World Health Organization BMI classification [22]. According to the World Health Organization BMI classification [22], BMI < 18.5, 18.5–24.9, 25.0–29.9, 30.0–34.9, 35.0–39.9, and ≥40.0 kg/m^2^ represent underweight, normal, overweight, obese class I, obese class II, and obese class III, respectively. Given the rarity of ≥40.0 kg/m^2^ in the South Korean population, obese class II (35.0–39.9) and obese class III (≥40.0) were combined into a single group, representing severe obesity (>35.0). The 30.0–34.9 (obese class I) group was considered the obesity group in this study. WC was divided into three groups (normal, high, and very high); normal, high, and very high WCs were <94 cm, 94–101.9 cm, and ≥102 cm in men and <80 cm, 80–87.9 cm, and ≥ 88 cm in women, according to the previous literature [23,24], respectively.

### 2.5. Endpoints

The primary endpoint was 30-day mortality, defined as any death within 30 days after hospital admission due to sepsis or septic shock. The secondary endpoint was 1-year mortality, defined as any death within 1 year after hospital admission due to sepsis or septic shock. The accurate death dates were followed up until April 2022, regardless of hospital discharge or transfer to long-term facility care centers.

### 2.6. Variables Analyzed

Demographic information, including sex and age at hospital admission with a diagnosis of sepsis, was collected. Socioeconomic status-related information such as employment status, household income level, and residence at hospital admission with a diagnosis of sepsis was obtained. The Charlson comorbidity index of the participants was calculated using ICD-10 codes (Table S1).

The insured were classified into five groups according to their household income: quartile ratios (Q1–Q4) and medical aid program groups. Level of household income, Q1–Q4, determines the insurance premiums of the insured; approximately 67% of their medical expenses are covered by the government. Extremely low-income populations (approximately 2.8%) are beneficiaries of the medical aid program, in which the government pays most of their medical expenses. Urban area for residence refers to Seoul and metropolitan cities, and rural areas refer to all other areas.

The type of hospital for sepsis was divided into four groups: general hospital, hospital, long-term facility care hospital, and others. We adjusted the treatment and care for sepsis, including admission to the ICU, ventilator support, extracorporeal membrane oxygenation support, or continuous renal replacement therapy use. Internal medicine as the department of admission and surgery-associated hospital admission were considered covariates.

### 2.7. Statistical Analysis

The Kolmogorov–Smirnov test was used to determine the normality of the distribution of continuous variables, including BMI and WC; it was confirmed that the continuous variables were not normally distributed. Thus, baseline characteristics are expressed as median values with interquartile ranges (IQR) and ranges for continuous variables and numbers of percentages for categorical variables.

The log odds of 30-day mortality or log relative hazard of 1-year mortality after sepsis according to pre-admission BMI or WC (continuous variables) were assessed using restricted cubic splines (RCS). The RCS examines the linear relationship between BMI and WC as continuous exposures with 30-day and 1-year mortality. Cox analysis was used to determine the log relative hazard for one-year mortality, while logistic analysis was applied to log odds for 30-day mortality [25].

Thereafter, we constructed a multivariable logistic regression model for 30-day mortality in patients with sepsis. All covariates were included in the model for adjustment, and results are expressed as odds ratios (ORs) with 95% confidence intervals (CIs). The goodness of fit of the multivariable model was determined using Hosmer–Lemeshow statistics. We also fitted a multivariable Cox regression model for 1-year mortality in patients with sepsis. The results are presented as hazard ratios (HRs) with 95% CIs, and log–log plots were used to confirm that the central assumption of Cox proportional hazard models was satisfied. No multicollinearity between variables was observed in multivariable models with a variance inflation coefficient criterion of <2.0. R software (version 4.0.3; R Foundation for Statistical Computing, Vienna, Austria) was used for statistical analyses. A *p*-value < 0.05 was considered statistically significant.

## 3. Results

### 3.1. Baseline Characteristics

**Figure 1:**
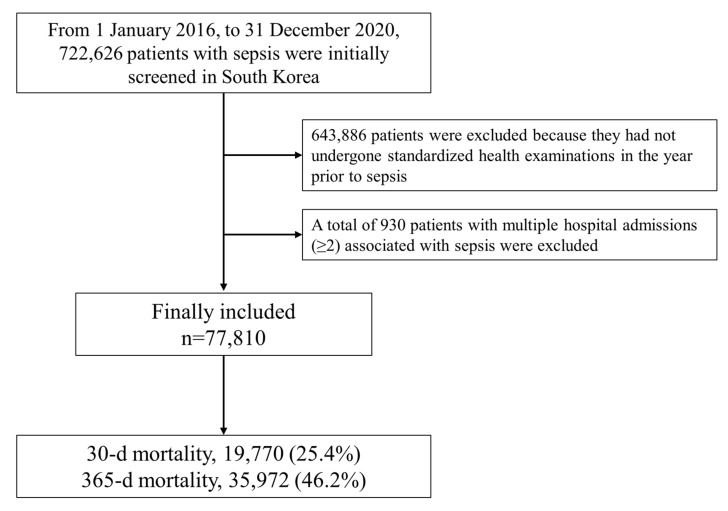
Flow chart depicting the patient selection process and mortality rate of the study population.

A flowchart of the participants’ selection and mortality rate of the study population is shown in Figure 1. From 1 January 2016 to 31 December 2020, 722,626 patients with sepsis were initially screened in South Korea. Among them, 643,886 patients were excluded because they had not undergone standardized health examinations in a year prior to sepsis. A total of 930 patients with multiple sepsis-related hospital admissions (≥2) were also excluded. Ultimately, 77,810 patients were included in this study. A total of 19,770 (25.4%) and 35,972 (46.2%) patients died within 90 days and 1 year after the date of sepsis diagnosis, respectively. The baseline patient characteristics are presented in Table 1. The median value of age was 68.0 years (IQR: 60.0–78.0 years, range: 40.0–84.0 years), and the proportion of men was 53.7% (41,790/77,810). The median values of BMI and WC were 23.4 kg/m^2^ (IQR: 21.0–26.0 kg/m^2^, range:10.0–54.5 kg/m^2^) and 90.5 cm (IQR: 79.6–102.5 cm, range: 58.1–131.3 cm), respectively.

### 3.2. Restricted Cubic Splines

**Figure 2:**
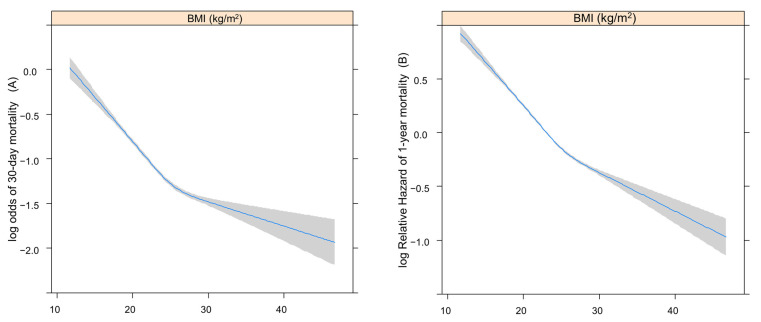
The RCSs present log odds of 30-day (**A**) or relative hazard ratio 1-year (**B**) mortality after sepsis, according to BMI. RCS, restricted cubic spline; BMI, body mass index.

**Figure 3:**
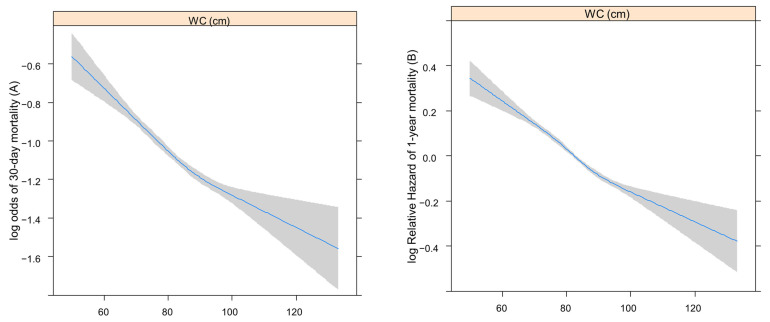
The RCSs present log odds of 30-day (**A**) or relative hazard ratio 1-year (**B**) mortality after sepsis, according to WC. RCS, restricted cubic spline; WC, waist circumference.

Figure 2 shows the RCSs that present the log odds of 30-day (A) or relative hazard ratio 1-year (B) mortality after sepsis according to BMI. Figure 3 shows the RCSs that present the log odds of 30-day (A) or relative hazard ratio 1-year (B) mortality after sepsis according to WC.

Both the log odds of 30-day and log relative hazard of 1-year mortality increased with lower BMI and WC.

### 3.3. Survival Analyses

Table 2 shows the 30-day mortality rate after sepsis. Using the multivariable logistic regression model, the underweight group showed an association with higher 30-day mortality (OR: 1.45; 95% CI: 1.36, 1.54, *p* < 0.001) compared with the normal BMI group. The overweight (OR: 0.80, 95% CI: 0.77, 0.84, *p* < 0.001) and obesity groups (OR: 0.85, 95% CI: 0.76, 0.95, *p* = 0.004) were associated with lower 30-day mortality than was the normal BMI group after adjusting for confounders including WC. However, the severe obesity group exhibited no significant differences compared with the normal BMI group (*p* = 0.068). Statistically significant increases in the 30-day mortality were observed in the high WC group (OR: 0.80, 95% CI: 0.77, 0.84, *p* < 0.001) and very high WC group (OR: 0.85, 95% CI: 0.76, 0.95, *p* = 0.004) compared with the normal WC group.

In the multivariable Cox regression model for 1-year mortality after sepsis (Table 3), the underweight group was associated with increased 1-year mortality compared with the group with a normal BMI (HR: 1.30, 95% CI: 1.26, 1.34; *p* < 0.001). Lower 1-year mortality was observed in the overweight (HR: 0.82, 95% CI: 0.80, 0.85, *p* < 0.001) and obesity groups (HR: 0.81, 95% CI: 0.76, 0.87, *p* < 0.001) compared with the normal BMI group. Compared with the normal WC group, the group with high WC (HR: 0.92, 95% CI: 0.89, 0.95, *p* < 0.001) and very high WC (HR: 0.89, 95% CI: 0.85, 0.93, *p* < 0.001) showed significant associations with lower 1-year mortality.

## 4. Discussion

Overweight and obese patients (25 ≤ BMI < 35) had lower 30-day and 1-year mortality, and underweight patients (BMI < 18.5) had higher 30-day and 1-year mortality after sepsis than those with normal BMI. The severe obesity group did not show any benefit or harm compared with the normal BMI group after adjusting for variables including WC. However, the high and very high WC groups showed lower 30-day and 1-year mortality after sepsis than did the normal WC group after adjustment for variables including BMI. This is the first study to reveal that abdominal obesity with a higher WC might be associated with a higher survival rate compared with normal WC.

Our finding is important because identifying high-risk patients with sepsis at hospital admission is important for providing intensive care. In addition, our finding is novel because pre-hospital admission WC has not been utilized as a mortality predictor in patients with sepsis. Prior studies have examined the impact of BMI or WC on mortality in patients with sepsis individually [7,9,10,26]. However, our study distinguishes itself by incorporating both variables into the model, thus providing a comprehensive analysis of the influence of BMI and WC on mortality in patients with sepsis. Therefore, we present the reasoning behind introducing this concept, which can be implemented in the future.

Previous studies using BMI-based obesity definitions have shown mixed results [7]. The first meta-analysis found that patients with sepsis who were overweight and obese (in Europe, Canada, USA, Australia, and Saudi Arabia) in the ICU were associated with decreased mortality [9]. Another meta-analysis found that compared with normal BMI, overweight (BMI, 25–29.9), but not obesity or morbid obesity, was associated with lower mortality in patients with sepsis (USA, Canada, and Europe) [26]. Each retrospective study showed different results regarding the obesity paradox of sepsis-related mortality.

In a few Asian studies, underweight patients had higher mortality rates than did normal or obese patients with sepsis [10,27,28,29]. China, Japan, and South Korea have fewer obese patients with sepsis compared with other countries [27,28,29,30]. In a study of patients in the USA, Canada, and Saudi Arabia, patients with BMI > 30 accounted for 29% of the population (5.8% with BMI > 40), whereas the Asian study included approximately 4% of patients with BMI > 30 [10,18,27,29,30]. A lower proportion of patients with obesity may have prevented the evaluation of the effect of obesity on sepsis-related mortality in patients with sepsis [10]. However, patients who were overweight and obese showed improved survival rates after sepsis compared with normal BMI patients. Moreover, underweight patients had higher mortality than normal BMI patients. However, this study included a similar proportion of obesity as other studies on East Asian patients.

The need for appropriate nutritional therapy for patients with sepsis is highlighted by the paradoxical fact that individuals with a low BMI (underweight group) have a greater mortality risk in sepsis. Appropriate nutritional assistance is a significant and contentious concern for patients suffering from sepsis [31], as this condition can trigger an immediate catabolic response that could result in the body’s energy reserves being broken down [32]. In patients with sepsis, a malnutritional state is a poor predictive predictor for mortality [33]. Nutritional supplementation is advised to address micronutrient and vitamin shortages and ensure that patients with sepsis receive enough protein [34].

Few studies have established a relationship between abdominal obesity (measured through WC) and sepsis-related mortality. Generally, a larger WC is associated with an increased risk of death, even after adjustment for BMI and other variables [35,36,37]. Almost all studies on the association between WC and sepsis-related mortality have reported similar results [38,39]. Even in studies showing lower sepsis-related mortality in those with high BMI or higher sepsis-related mortality in those with low BMI, a larger WC is associated with higher sepsis-related mortality [27,40].

Wang et al. defined obesity by WC >102 cm in men, >88 cm in women, or BMI ≥30 mg/cm^2^. They also revealed that obesity was related to lower sepsis-related mortality during a 6-year observation period after sepsis [16]. However, whether a larger WC was beneficial to patients with sepsis could not be ascertained because the authors did not distinguish abdominal obesity from high BMI.

A larger WC may be considered a more serious health problem than BMI. A person with high muscle mass and low adipose tissue may be healthy with having a high BMI and smaller WC [41]. The cardiometabolic status may be worse in Asia than in other ethnicities with the same WC, a surrogate of visceral adipose tissue, because Asians have less subcutaneous abdominal tissue than others [17]. In a retrospective study of a population-based multicenter prospective cohort in China, Weng et al. found that a higher WC was associated with increased sepsis-related mortality despite the fact that overweight and obesity were not associated with lower sepsis-related mortality in the same study [10]. Unlike previous studies, we showed herein, for the first time, that a higher WC was associated with lower sepsis mortality using East Asian data.

The mechanism underlying the obesity paradox has not been clarified. One possible reason might be that adipose tissue could act as a great energy reservoir and prevent the wasting of muscular tissue during the catabolic crisis of sepsis and septic shock despite obesity-related harm. The catabolic crisis of sepsis is accompanied by lower nutritional support because low-calorie nutrition is recommended for critically ill patients during the early sepsis period [42]. Unstable vital signs, high-dose vasopressors, dysfunction of the liver or kidney, intestinal fistula, or leakage could interrupt adequate nutritional support [43]. In addition, obesity has the following advantages over underweight or normal weight: preconditioning of inflammation, immune system anti-inflammatory profile, neutralization of endotoxins, and steroid synthesis [7,43,44,45].

This study has certain limitations. First, we could not include information missing from the NHIS database. Detailed laboratory data, imaging data, and charts were not included. Second, since we used BMI/WC data from standardized health examinations a year before sepsis, the duration from the date of BMI/WC measurement to sepsis onset could have ranged between 1 day and 2 years. Therefore, there might have been some changes between BMI/WC measurement and hospital admission due to sepsis. Lastly, we could not include patients who did not undergo standardized health examinations despite being covered by the government. Considering that individuals aged ≤ 39 years did not undergo the standard health examination and that there was a 50% chance of participation in a certain year (i.e., the year prior to sepsis) and 70–80% participation rate in the standard health examination, approximately 25–30% of patients with sepsis could have been included in this study. However, approximately 10.8% of patients with sepsis were finally included in this study. Since age, disability, and socioeconomic status were reported to affect the participation rate in the standard health examination, there might be selection bias in this study, which should be carefully considered while interpreting results [21].

## 5. Conclusions

Patients with abdominal obesity and overweight and obesity (BMI 25–34.9 kg/m^2^) and with sepsis showed reduced mortality, whereas underweight (BMI < 18.5 kg/m^2^) patients with sepsis in South Korea showed increased mortality. Our study’s findings indicate that patients with sepsis who are underweight or malnourished have a higher mortality rate, indicating the critical importance of proper nutrition care. This study also showed that an increase in excess weight or adipose tissue was linked to a lower patient mortality rate in specific circumstances, such as sepsis. Future research should focus on the nutritional status of each patient admitted with sepsis and nutritional interventions and treatments to improve outcomes for patients with sepsis.

## Figures and Tables

**Table 1:** Baseline patient characteristics.

Variable		Median [IQR, Range] or *N* (%)
Age, year		68.0 [60–78, 40–84]
Male sex		41,790 (53.7)
Main diagnosis of sepsis		27,197 (35.0)
Having a job		46,264 (59.5)
Residence at sepsis		
	Urban area	25,353 (32.6)
	Rural area	52,457 (67.4)
Household income level		
	Medical aid program	4960 (6.4)
	Q1 (lowest)	13,618 (17.5)
	Q2	11,852 (15.2)
	Q3	15,542 (20.0)
	Q4 (highest)	24,787 (31.9)
	Unknown	7051 (9.1)
BMI, kg/m^2^		23.4 [21.0–26.0, 10.0–54.5]
	<18.5	6386 (8.2)
	18.5–24.9	46,254 (59.4)
	25.0–29.9	21,419 (27.5)
	30.0–34.9	3221 (4.1)
	>35.0	530 (0.7)
Waist circumference		90.5 [79.6–102.5, 58.1–131.3]
	Normal	51,259 (65.9)
	High	15,804 (20.3)
	Very high	10,747 (13.8)
CCI, point		3.0 [1.0–4.0, 0–17]
ICU admission		24,413 (31.4)
Ventilator support		12,682 (16.3)
ECMO support		315 (0.4)
CRRT use		3522 (4.5)
Type of hospital		
	General hospital	55,873 (71.8)
	Hospital	7324 (9.4)
	Long-term facility care hospital	14,290 (18.4)
	Other	323 (0.4)
IM department		52,304 (67.2)
Surgery-associated hospital admission		30,808 (39.6)
Year		
	2013	7213 (9.3)
	2014	7277 (9.4)
	2015	7923 (10.2)
	2016	9271 (11.9)
	2017	10,958 (14.1)
	2018	11,415 (14.7)
	2019	11,719 (15.1)
	2020	12,034 (15.5)

BMI, body mass index; CCI, Charlson comorbidity index; ICU, intensive care unit; ECMO, extracorporeal membrane oxygenation; CRRT, continuous renal replacement therapy; IM, internal medicine.

**Table 2:** Multivariable logistic regression model for 30-day mortality.

Variable		OR (95% CI)	*p*-Value
Age, year		1.04 (1.04, 1.04)	<0.001
Male sex		1.38 (1.32, 1.44)	<0.001
Main diagnosis of sepsis		1.03 (0.99, 1.07)	0.193
Having a job		0.93 (0.90, 0.97)	0.001
Residence at sepsis			
	Urban area	1	
	Rural area	1.06 (1.02, 1.10)	0.007
Household income level			
	Medical aid program	1.06 (0.98, 1.16)	0.159
	Q1 (lowest)	1	
	Q2	0.93 (0.87, 0.99)	0.021
	Q3	0.92 (0.86, 0.97)	0.003
	Q4 (highest)	0.85 (0.80, 0.89)	<0.001
	Unknown	0.67 (0.62, 0.73)	<0.001
BMI			
	<18.5	1.45 (1.36, 1.54)	<0.001
	18.5–24.9	1	
	25.0–29.9	0.80 (0.77, 0.84)	<0.001
	30.0–34.9	0.85 (0.76, 0.95)	0.004
	>35.0	0.78 (0.59, 1.02)	0.068
Waist circumference			
	Normal	1	
	High	0.89 (0.84, 0.93)	<0.001
	Very high	0.87 (0.81, 0.94)	<0.001
CCI, point		1.10 (1.08, 1.12)	<0.001
ICU admission		0.93 (0.88, 0.98)	0.008
Ventilator support		6.49 (6.14, 6.86)	<0.001
ECMO support		1.26 (0.99, 1.60)	0.066
CRRT use		2.65 (2.43, 2.88)	<0.001
Type of hospital			
	General hospital	1	
	Hospital	1.74 (1.64, 1.86)	<0.001
	Long-term facility care hospital	1.59 (1.50, 1.68)	<0.001
	Other hospital	0.93 (0.65, 1.31)	0.661
IM department		1.27 (1.22, 1.33)	<0.001
Surgery-associated hospital admission		1.01 (0.96, 1.06)	0.695
Year			
	2013	1	
	2014	0.96 (0.89, 1.04)	0.337
	2015	0.91 (0.84, 0.99)	0.019
	2016	0.85 (0.78, 0.91)	<0.001
	2017	0.75 (0.70, 0.81)	<0.001
	2018	0.71 (0.66, 0.76)	<0.001
	2019	0.70 (0.65, 0.80)	<0.001
	2020	0.75 (0.70, 0.80)	<0.001

OR, odds ratio; CI, confidence interval; BMI, body mass index; CCI, Charlson comorbidity index; ICU, intensive care unit; ECMO, extracorporeal membrane oxygenation; CRRT, continuous renal replacement therapy; IM, internal medicine.

**Table 3:** Multivariable Cox regression model for 1-year mortality.

Variable		HR (95% CI)	*p*-Value
Age, year		1.03 (1.03, 1.03)	<0.001
Male sex		1.37 (1.33, 1.40)	<0.001
Main diagnosis of sepsis		0.92 (0.90, 0.94)	<0.001
Having a job		0.94 (0.92, 0.96)	<0.001
Residence at sepsis			
	Urban area	1	
	Rural area	1.05 (1.03, 1.07)	<0.001
Household income level			
	Medical aid program	1.08 (1.03, 1.14)	0.002
	Q1 (lowest)	1	
	Q2	0.96 (0.93, 0.99)	0.040
	Q3	0.96 (0.93, 0.99)	0.017
	Q4 (highest)	0.91 (0.89, 0.94)	<0.001
	Unknown	0.87 (0.83, 0.91)	<0.001
BMI, kg/m^2^			
	<18.5	1.30 (1.26, 1.34)	<0.001
	18.5–24.9	1	
	25.0–29.9	0.82 (0.80, 0.85)	<0.001
	30.0–34.9	0.81 (0.76, 0.87)	<0.001
	>35.0	0.93 (0.79, 1.09)	0.357
Waist circumference			
	Normal	1	
	High	0.92 (0.89, 0.95)	<0.001
	Very high	0.89 (0.85, 0.93)	<0.001
CCI, point		1.08 (1.06, 1.09)	<0.001
ICU admission		0.90 (0.87, 0.93)	<0.001
Ventilator support		3.43 (3.32, 3.54)	<0.001
ECMO support		1.18 (1.04, 1.35)	0.010
CRRT support		1.74 (1.67, 1.82)	<0.001
Type of hospital			
	General hospital	1	
	Hspital	1.42 (1.37, 1.48)	<0.001
	Long-term facility care hospital	1.87 (0.81, 1.94)	<0.001
	Other	0.66 (0.52, 0.86)	0.002
IM department		1.23 (1.20, 1.26)	<0.001
Surgery-associated hospital admission		1.17 (1.14, 1.21)	<0.001
Year			
	2013	1	
	2014	1.00 (0.96, 1.05)	0.888
	2015	0.93 (0.89, 0.97)	0.001
	2016	0.89 (0.85, 0.93)	<0.001
	2017	0.83 (0.79, 0.87)	<0.001
	2018	0.80 (0.76, 0.83)	<0.001
	2019	0.79 (0.76, 0.83)	<0.001
	2020	0.81 (0.78, 0.84)	<0.001

HR, hazard ratio; CI, confidence interval; BMI, body mass index; CCI, Charlson comorbidity index; ICU, intensive care unit; ECMO, extracorporeal membrane oxygenation; CRRT, continuous renal replacement therapy; IM, internal medicine.

## Data Availability

Data will be available upon reasonable request to the corresponding author.

## Supplementary Materials

The following supporting information can be downloaded at https://www.mdpi.com/article/10.3390/diagnostics14060574/s1: Table S1. The ICD-10 codes used to compute the Charlson comorbidity index.
